# Recipients’ Experiences after Organ Transplantation

**Published:** 2018-05-01

**Authors:** Z. Sheikhalipour, V. Zamanzadeh, L. Borimnejad, L. Valizadeh, M. Shahbazi, A. Zomorrodi, M. Nazari

**Affiliations:** 1 Medical-Surgical and Operating Room Department, Nursing and Midwifery School, Tabriz University of Medical Sciences, Tabriz, Iran; 2 Pediatric Nursing Department, Nursing and Midwifery School, Iran University of Medical Sciences, Tehran, Iran; 3 Pediatric Nursing Department, Nursing and Midwifery School, Tabriz University of Medical Sciences, Tabriz, Iran; 4 Behavioral Health Promotion and Education/Public Health, Jackson State University, Jackson, MS 39217, USA; 5 Urology Department, Faculty of Medicine, Tabriz University of Medical Sciences, Tabriz, Iran; 6 Theology Department, Faculty of Theology & Islamic Science, Tabriz University, Tabriz, Iran

**Keywords:** lived experiences, phenomenological study, hermeneutic, transplantation, nurse, post-transplantation

## Abstract

**Background:**

After organ transplantation, many patients have diverse experiences; they face many changes in the physical and emotional aspects of their life. Patients’ understandings of the post-transplantation period influence their adaptation to the changes. There is a need to improving the knowledge of patients’ unique experiences of post-transplantation period and the changes occur in their life.

**Objective:**

To explore the experiences of organ recipients in the post-transplantation period.

**Methods:**

In a qualitative research using a hermeneutical phenomenological approach, data were collected from April 2015 to June 2016. Participants were consisted of 15 patients who received organ chosen using a purposive sampling method. In-depth semi-structured interviews were held with them. The collected data were analyzed using Diekelmann’s hermeneutical analysis approach.

**Results:**

The data analyses led to the development of 3 main themes and 17 subthemes as “back from the grave” with the subthemes of “organ as the God’s deposit,” “God as the source of life,” and “new life”; “chapter of prosperity” with the subthemes of “the spring of the body,” “recovery,” “peace and joy,” “benevolent and good behavior,” “renewal,” “opportunity of being together again,” “golden age,” “positive perspective,” “the sense of normality,” “the return of health,” and “spiritual evolution”; and “the fall” with the subthemes of “a lack of energy,” “the mirage of transplantation,” and “hell on the earth.”

**Conclusion:**

The patients had diverse experiences of the post-transplantation period, which varied from the feeling of exhilaration and youth to losing energy and the wish for not undertaking organ transplantation.

## INTRODUCTION

Patients have various experiences in the post-transplantation period; they face many changes in the physical and emotional aspects of their life during this period [1, 2]. While patients expect full recovery following the transplantation, soon they understand that they have recovered from a chronic disease, but should adapt with another health issue [3]. In other words, they should fight for survival both before and after the transplantation [4].

Some patients encounter numerous surgical and medical problems after organ transplantation; all of them experience medications side-effects and consequences of severe medical regimen [5]. Moreover, they are at the risk of organ rejection and need to cope with a range of problems such as life changes, drugs, and family and job issues [6]. Depending on patients’ psychological conditions, they may have different experiences and understandings of the post-transplantation period, which will set their adaptation level with changes during this period. Therefore, awareness of patients’ understandings and experiences by nurses and other health care providers is useful to improve the quality of care and facilitate their adaptation to health-related issues by health care professionals [7]. Also, such understandings help with the designation of appropriate health improvement interventions by health care providers [8]. In this respect, nursing care is benefited from an in-depth understanding of patients’ perspectives through providing information about how to create an appropriate environment for fast recovery.

In fact, several studies have so far assessed organ transplant outcomes and related measures, including health-related quality of life (HRQoL) [9], organ integration, and public perception [10], and psychosocial variables and adherence [5]. Multiple meta-analyses have been conducted to develop a better understanding of the organ transplant process and determine which of these (and other) factors affect long-term outcome of organ recipients [11]. However, the field of organ transplantation is rapidly changing, so the way of understanding and experiencing transplantation phenomenon would be different at each time. Moreover, the influence of psychological factors on outcomes is just the beginning to be understood. 

Qualitative research is the most appropriate method for understanding patients’ experiences of the transplantation process [12]. Of the available qualitative methods, phenomenology is a suitable method of data collection and analysis for exploring the essence of patients’ experiences and meanings. This method also has a holistic approach to phenomena rather than a focused one [13]. Therefore, this study was conducted to explore the experiences of organ recipients of the post-transplantation period. The unique experience of receiving a transplanted organ and the consequent changes in life demand understanding of the experiences of these patients. This understanding can provide a deep insight that is crucial for health care providers to help patients during clinical practice. The findings of this study can help nurses devise strategies that help patients easily pass through the transplantation period and cope with its health-related issues. 

## MATERIALS AND METHODS

Design 

This study sought a hermeneutic phenomenological design to find the answer to the following question: “What are patients’ experiences of the post-transplantation period?” Since the hermeneutic phenomenological method encompasses both description and interpretation. Therefore, in this study, in addition to obtaining the description of organ recipients’ experiences, the interpretations of the meanings behind the descriptions were also sought.

Setting and Participants 

Given the principle of maximum variation in sampling and to ensure data collection richness, different places and participants were considered. Patients undergoing heart, kidney, and liver transplantation were recruited in this study. Research sites were the heart clinic in a large teaching hospital affiliated to Tehran University of Medical Sciences, Tehran, where patients would be visited by physicians every two months, and the kidney transplantation ward affiliated to Tabriz University of Medical Sciences, Tabriz, where patients would be monitored for post-transplantation complications. Also, the Association of Liver Transplantation affiliated to Tabriz University of Medical Sciences, was found a suitable place for data collection, because many patients after transplantation were referred there for seeking financial assistances. Lastly, the researcher referred to the emergency department of a teaching hospital affiliated to Tabriz University of Medical Sciences to recruit blood recipients for various reasons such as anemia. 

Ethics

The research proposal was approved by the Ethics Committee of Tabriz University of Medical Sciences, Tabriz, Iran (decree number: TBZMED.REC.1394.167). Participants were informed of the study’s method and process. They were also ensured of anonymity and confidentiality of the data collection process. It was stated that the collected data will only be used for research purposes and shared among the research team members and each person was given a number to protect their anonymity. The permission to tape-record the interviews was obtained from the participants. Lastly, the participants were asked to sign a written informed consent.

Data Collection

Data were collected from April 2015 to June 2016. In this study, 15 organ recipients were recruited. They were consisted of three liver, four kidney, and eight heart recipients (Table 1). In-depth semi-structured interviews were held in times and places convenient for them. The main questions asked were “what is your feeling, when you hear the word of ‘organ transplantation’?” and “what is your experiences of organ transplantation?” 

**Table 1:** The demographic characteristics of the participants

Participant #	Age	Sex*	Education level	Job	Marital status	Residency place	Type of transplantation	Time after transplantation†
1	49	F	Associate Degree	Housewife	Married	Tabriz	Liver	8 y
2	57	F	Diploma	Housewife	Married	Tabriz	Liver	2 y and 6 m
3	46	M	Master degree	Employee	Married	Tabriz	Liver	1 y
4	42	M	Diploma	labor	Married	Osko	Heart	3 y and 6 m
5	57	M	Diploma	Businessman	Married	Tehran	Heart	1 y and 4 m
6	57	M	Diploma	Businessman	Married	Karaj	Heart	5 y
7	53	M	Diploma	Taxi driver	Married	Tehran	Heart	5 y and 6 m
8	55	F	Diploma	Housewife	Married	Azarshahr	Kidney	5 y and 3 m
9	34	F	Illiterate	Housewife	Single	Kaleibar	Kidney	9 m
10	34	F	Diploma	Housewife	Married	Tabriz	Kidney	First transplantation 12 y ago and Second one 4 m
11	27	F	Diploma	Housewife	Married	Bojnord	Heart	1 year
12	68	M	Diploma	Businessman	Married	Sari	Heart	3 y and 6 m
13	29	F	Diploma	Housewife	Married	Tehran	Heart	2 y and 3 m
14	40	M	Diploma	Labor	Married	Guilan	Heart	2 y and 6 m
15	28	M	Diploma	Carpet weaver	Married	Tabriz	Kidney	3 y

* F: Female, M: Male;

† y: year, m: month

Since four participants were interviewed twice, in total 19 sessions of interviews were held. The interviews lasted between 15 and 120 minutes. The interviews were carried out in a quiet and private place at the research sites, except for two participants who preferred their own homes.

Data Analysis and Rigor

The collected data were analyzed using the Diekelmann’s hermeneutical analysis approach. This method has seven stages: (1) examining the text as a whole, (2) summarizing sections of the text and identifying categories, (3) analyzing the text based on categories in step two, (4) identifying relational themes in the text, (5) generating constitutive patterns in the text, (6) validating the analysis by persons not part of the research team but familiar with both textual content and the research method, and (7) preparing the final report using sufficient quotations from the interview to allow for validation of the findings by the reader [14]. 

By using this method, first the interviews were transcribed verbatim and read by the research team members to gain a general understanding of the text. The researchers wrote interpretive notes and share together and held discussions to compare their findings in terms of similarities and differences. Disagreement in interpretation was resolved by returning to the text. In some cases, the participant was contacted for clarification. Also, interpretive summaries of each interview was given to participants to ensure that our interpretation accurately reflected their experiences of the study phenomenon. The principal investigator wrote a combined analysis of each text. Through comparing and contrasting texts, the composite analyses themes that repeated and mirrored the shared practices and common meanings were identified and described. This description was presented to the team and discussed. Next, associated patterns with the developed themes were identified. Lastly, the themes and patterns along with a summary of the interviews transcriptions were given to the research team members and five other qualitative research experts to provide feedbacks on the analysis process. Responses and suggestions were integrated into the final draft. Anything that was considered to be unsupported in the text was deleted. 

## RESULTS

The data analyses led to the development of three main themes as “back from the grave” with the subthemes of “organ as the God’s deposit,” “God as the source of life,” and “new life”; “chapter of prosperity” with the subthemes of “the spring of the body,” “recovery,” “peace and joy,” “benevolent and good behavior,” “renewal,” “opportunity of being together again,” “golden age,” “positive perspective,” “the sense of normality,” “the return of health,” and “spiritual evolution”; and “the fall” with the subthemes of “a lack of energy,” “the mirage of transplantation,” and “hell on the earth.”

Back from the Grave

In the pre-transplantation period, the participants were so scared of death during the transplantation surgery and saw themselves one step away from the death. Therefore, undergoing the transplantation was described as death and resurrection.

*Organ as the God’s Deposit*

The participants stated that the transplanted organ was a great blessing granted by God. They managed to find an organ by the divine knowledge. Therefore, God granted them a new life through the provision of the possibility of undergoing transplantation. 

“When I came to consciousness, I found what a great blessing was given to me by God. By the divide decree, I was chosen as the person to undergo organ transplantation.” (P 3)

*God as the Source of Life*

The participants knew that it was likely to die during the transplantation operation. Since undergoing the organ transplantation was inevitable, they put their trust in God and prayed for to be saved. Therefore, they thanked God when they found themselves alive after the surgery and believed that God breathed the spirit again into their body. 

“I put my trust in God and accepted undergoing the surgery…it is a great blessing that God has given me back to this world.” (P 4)

*New Life*

It was mentioned that the soul was inspired again into their body and they were reborn and a new life has begun.

“I felt that a new life has been started…after the surgery, I thought that a new life has been given to me. I thought that I have been reborn.” (P 1) 

Chapter of Prosperity

The participants experienced a new chapter of their life in the post-transplantation period that was described as the season of flourishing full of vitality and calm.

*Spring of the Body*

After organ transplantation, they felt being young and full of the youth power. Also, with the arrival of organ to their body, a new life in their bodies was started and their body was full of energy and vitality.

“After transplantation, I feel young in my body. I felt I was too young.” (P 5)

“I felt that I was young. I was 50 years old, but I felt I was 25 years old.” (P 8)

*Recovery*

They stated that after transplantation, they felt much power and energy and were able to do anything.

“My body has been changed. I have become smart now. I could not work too much before the transplantation, but I do whatever I want without becoming tired.” (P 10)

*Peace and Joy*

The patients declared that after organ transplantation they felt relaxed. They were happy that did not suffer from the disease anymore and continued living with hope.

“Overall, I became hopeful to life. Before the transplantation, I went to a coma for several times, but after the transplantation, I felt relaxed and did not suffer anymore.” (P 2)

*Benevolent and Good Behavior*

After organ transplantation, positive changes occurred in the participants’ thoughts and behavior. For instance, they had become optimist toward life, become patient in dealing with life issues, and become more emotional in communication with others, especially family members. 

“I have positive thoughts and I feel that I am in peace and patiently follow my problems to be solved. I have become quite soft and put aside my anger and deal with my wife pretty well.” (P 6)

*Renewal*

They believed that the ill organ has been replaced by a healthy one. Therefore, they had the feeling of renewal and vitality in their bodies. They stated that they began their new life with something new.

“I have various feelings. For instance, I feel my own organ has been damaged and now a new one is available in my body. This new organ gives me a new life.” (P 1)

*Opportunity of Being Together Again*

The participants were away from their family members and children due to illness and frequent hospitalization. After the transplantation, they had more time to spend with them, and therefore were happy. They were worried about death during surgery and leaving their family members for being hospitalized. Therefore, they were very happy for becoming able to visit their family members. 

“I waited for nine months before undergoing organ transplantation. I was away from home and hospitalized in another city. Every moment, I wished I had been near my children. Such feelings influenced me so much. When we got together, I felt so happy and did my best to make my family satisfied.” (P 2)

*Golden Age*

The participants described their life after the transplantation as a fruitful period. They benefited from organ transplantation, enjoyed working, knew the worth of their life moments and used their time to do good things. They set goals for their life and through increasing their activities tried to achieve them. 

“This is one year passed after I underwent organ transplantation and it seems that it has been 100 years. I have benefited a lot from my family members, children, my work, and even I passed the doctoral degree entrance exam and all these happened only in one year.” (P 3)

*Positive Perspective*

The participants described organ transplantation as a beautiful experience. They were satisfied with it and had positive perspective toward it. 

“Organ transplantation is an excellent procedure. I advise patients to undergo this procedure. Its consequence is satisfying. This is what I experienced.” (P 7)

*The Sense of Normality*

The recipients were released from all limitations imposed on them by the disease and felt like a normal person with regard to increasing their level of activities. 

“I am thinking about the moments that I was unable to walk, but I can walk and even run. I run similar to others. I am proud of myself that I can go to the park for doing exercise like others.” (P. 4)

*The Return of Health*

Organ transplantation meant the return of health and wellbeing. The participants regained their health through transplantation and were free from suffering. 

“Organ transplantation means wellbeing. It is much better than dialysis. I tolerated hemodialysis for nine months and experienced headache after each session of hemodialysis. I could not even talk and soon after a new session of hemodialysis would be started. Now, I am OK and experience no headache at all.” (P 9)

*Spiritual Evolution*

They made a better relationship with God after transplantation and felt so close to the Lord. They aimed to do good deeds and carryout charity activities.

“I have tried to do good deeds. I respect my religious rituals and practice them. I make connection with God to send an acceptable file of actions to God.” (P 3)

The Fall

The patients entered a new stage of life the so-called “the fall.” In this stage, medication side effects appeared and disease-related issues led to the development of physical and psychological problems. Moreover, they surrounded to the feeling of lack of energy and losing vitality and youth power. 

*A Lack of Energy*

Over time, the feeling of vitality and youth power was diminished and was replaced by the feeling of weakness and fatigue.

“After transplantation I felt that I was young and could do anything, but now I think that I have no energy. My extremities’ edema has made me so tired.” (P 8)

*The Mirage of Transplantation*

Soon after the transplantation, the participants wished for not undergoing this procedure. They believed that no difference was present between the pre- and post-transplantation periods and organ transplantation was in vain.

“I experience a lot of problems for post-transplantation drugs. Sometimes, I think that I would not have undergone this procedure at all. Many restrictions have been imposed on my life…laboratories tests, medications, so on. I have no passion to transplantation anymore. It seems that I tolerate unnecessary sufferings. I have been tired of my life.” (P 1)

*Hell on the Earth*

It was mentioned that the post-transplantation period was like living in the hell. They had taken the responsibility of taking care of the transplanted organ. Also, following the imposed restrictions and regimens and bad financial conditions led to the feeling of humiliation and being tormented.

“Organ transplantation is our mourning and justice…I am not satisfied. I cannot take the responsibility. I suffer...” (P 6) 

## DISCUSSION

According to the findings of this study, the patients had various perspectives and experiences of the post-transplantation period from the sense of strength and youth to the loss of power and wish for having not undergone organ transplantation. Our findings were presented by three themes of “back from the grave,” “chapter of prosperity,” and “the fall.” 

Those patients that saw themselves one step away from the death prior to the transplantation experienced that the soul was inspired to their body and returned to life. They were reborn and entered a new life, and believed that organ transplantation was the divide blessing. Similarly, the findings of a study by Peyrovi (2014) showed that patients believed in God’s willing in organ transplantation and sought help from the great grace of God [8]. In another study, many participants believed that their life was in debt to God [15]. However, Sadala reported that organ transplantation was only an incidental event in their life [4]. The results of a systematic review on organ transplantation showed that patients would consider themselves lucky for undergoing transplantation [16]. In our study, the participates’ perspectives of organ transplantation stemmed from their religious beliefs. From Iranian Muslims’ perspectives, people’s life is in God’s hands and nothing except God can change a person’s fate. Therefore, our participants believed in Gods’ willingness and help for the provision of appropriate organs for donation to them. 

On the other hand, the participants also experienced the best moments of their life in the post-transplantation period. The presence of a new organ in their body led to emerging the feeling of joy and happiness. They felt relaxed, became optimist toward life, and became patient in dealing with life issues. The results of Tayebi’s study showed that the first phase after organ transplantation is celebration and happiness from patients’ perspectives [17]. Another study showed that in spite of patients’ knowledge of the risk of organ rejection, drug dependencies, changes in the body conditions, they show happiness and celebrate the being alive [12]. According to our findings, the major reason for changes in the patients’ physical and psychological conditions was the disappearance of disease-related signs and symptoms. Therefore, the recipients were happy despite of imposed restrictions due to the post-transplantation regimens. By the way, patients’ happiness was not for having no knowledge of health-related issues during the post-transplantation period. The results of the study by Stolf showed that too much optimism of patients toward transplantation and lack of knowledge of limitations of organ transplantation might be barriers to following up the treatment modalities [12]. Therefore, the assessment of patients’ knowledge of the transplantation process and provision of information to patients are suggested. 

The patients felt a new life after transplantation and believed that the new organ gave them a new life. According to previous studies, patients passed through various phases after surgery the so-called “phases of toleration to the disease.” Facing the disease and reformation occurred after transplantation [18]. However, according to the study by Kaba, patients found it difficult to accept the new organ in their body and found the replacement of the old organ by a new one as a conflicting experience [15]. In spite of the presence of such a conflict, our study participants were happy that a sick organ was replaced by a healthy one, which could influence coping with the changes in the post-transplantation process. 

It was found that the patients had more time to spend with their family members and children in the post-transplantation period comparing with the past. After the transplantation, they happily spend their time to meet their family members’ needs. Similar studies described the recovery of social and familial connections soon after transplantation [17]. 

The patients experienced a fruitful period of life after transplantation, returned to work, increased the level of their activities and enjoyed their life. Similar studies confirm patients’ abilities to return to normal life and recover from the disease, improve spiritual well-being and enjoy life after transplantation [19]. 

In addition, the participants made a close connection with God through praying and showing their gratitude for being granted a new life after the transplantation. As one part of the findings of Peyrovi’s study, “the significant role of God in life” indicates a strong connection and belief of patients created after transplantation in response to God’s favor for granting them a new life [8]. Also, the role of religious beliefs and their role in adaptation to post-transplantation challenges have been emphasized [8, 20, 21]. It is noted that Iranians’ religious beliefs regarding the role of God and its supportive role in people’s life encourage them to make a connection to God through praying and asking for his support in their life. Also, they show their gratitude to God through continuous praying. 

Our study participants achieved a normal life after transplantation and were able to follow their routine activities. Corbin reported that patients described well-being as having no bizarre experience, no pain and suffering, and a healthy body [22]. One of the main categories developed by the Tong’s study was the strong familial support indicating that patients believed to be normal and healthy, did not want to be supported too much and loss their independence [16]. Another study on adolescents found that patients were concerned with not being seen normal by their colleagues [23]. In spite of taking medication, the patients took their life control and returned to normal activities in order to feel normal.

According to some participants’ experiences, they lost their youth power and happiness after transplantation, and wished for not being undergone transplantation. In line with our findings, Simons believed that patients achieved a great success in life, though they knew being caught with a chronic disease that needed a long-life treatment regimen [24]. Over time, patients’ concern with organ rejection and drugs’ side effects lead to getting tired of the treatment process and indicate the need for nursing intervention to help patients with effective adaptation. 

The imposed restrictions and regimens and bad financial conditions led to the feeling of humiliation and being tormented among the participants. Other studies have indicated depression and psychological issues [11] as well as patients’ suffering after organ transplantation [25].

One limitation of the current study was that people from all religions and tribes in Iran were not studied. Because people with different religions and cultures may have altered experiences after transplantation, it is suggested that future studies consider this variable.

In conclusion, the patients had diverse experiences of the post-transplantation period, which varied from the feeling of exhilaration and youth to losing energy and the wish for not undertaking organ transplantation. Therefore, nursing interventions are required to help patients with cope with drugs’ side effects and health-related issues in the post-transplantation period. Home care services have not been established in Iran and patients need to receive information from physicians that are mainly limited to the medication process. Therefore, there is a need to the development of holistic post-transplantation services delivered by nurses in order to create happy moments after organ transplantation. 
